# A Forecasting Model for Feed Grain Demand Based on Combined Dynamic Model

**DOI:** 10.1155/2016/5329870

**Published:** 2016-09-06

**Authors:** Tiejun Yang, Na Yang, Chunhua Zhu

**Affiliations:** School of Information Science and Engineering, Henan University of Technology, Zhengzhou 450001, China

## Abstract

In order to improve the long-term prediction accuracy of feed grain demand, a dynamic forecast model of long-term feed grain demand is realized with joint multivariate regression model, of which the correlation between the feed grain demand and its influence factors is analyzed firstly; then the change trend of various factors that affect the feed grain demand is predicted by using ARIMA model. The simulation results show that the accuracy of proposed combined dynamic forecasting model is obviously higher than that of the grey system model. Thus, it indicates that the proposed algorithm is effective.

## 1. Introduction

The grain used in feeding is the second largest grain used in China; its quantity and proportion of the total grain consumption grow stably. It is of great significance to ensure food security in our country by exploring the changes of feed grain demand and its influencing factors. However, the special research of China's feed grain demand is scattered, which lacks objective statistics and always exists in projections of the total grain consumption. The forecasting methods of feed grain demand in existing literature can be divided into two kinds: one is using some quantitative methods such as time series regression, model of consumer demand system, and farming grain consumption, based on the analysis about the situation of the feeding food consumption over the past few years to analysis and forecast [1, 2]; the other is from the perspective of nutrition standards analysis of meat, eggs, milk, per capita consumption of aquatic products to predict the future demand for animal products and then use the ratio of feed to meat (i.e., the conversion rate of feed grains) to predict the feed grain demand [3, 4]. Actually, the feed grain demand is affected by population growth, urbanization level, per capita income (urban residents per capita income and rural ones per capita income), and other factors [5, 6], which suggest that there should be a comprehensive survey about correlation degree between the feed grain demand and its influence factors for improving the prediction accuracy, and the corresponding prediction model should be generalized. In this paper, the correlation coefficients of feed grain demand and its influence factors are calculated quantitatively on the basis of the second kind of forecasting method; then the major factors have been chosen; finally the dynamic prediction of influence factors and feed grain demand can be realized by using the ARIMA model and multiple regression model, respectively.

## 2. Relational Coefficient Analysis of Influence Factors to Feed Grain Demand

### 2.1. Grey Relational Analysis

The essence of grey relational degree is to make a geometric comparison in the data series which are responded to the changing characteristics of all factors. The closer the curves are, the greater the relational grade of the corresponding series is and vice versa. The use of the grey relational analysis can define the changing trend of all factors in this system and find out the main factors which affect the further development of the system so as to grasp the main features of things and the principal contradiction, promote, and guide the system to rapid, health, and efficient development [7]. The basic steps of grey relational analysis are as follows.

**Step 1 .**

Assume that the reference sequence is *x*_0_(*k*) and related comparison sequences are *x*_*i*_(*k*). They are expressed as *x*_0_(*k*) = {*x*_0_(1), *x*_0_(2),…, *x*_0_(*n*)} and

(1) $$\begin{array}{c} x_{i}\left(\;k\;\right)=\left\{\;x_{i}\left(\;1\;\right),x_{i}\left(\;2\;\right),\ldots ,x_{i}\left(\;n\;\right)\;\right\},\;\left(i=1,2,\ldots ,m\right). \end{array}$$

**Step 2 .**

Dis-dimension treatment to the data sequence [8]. Here, we illustrate the initiating. Then it can get the reference sequence *y*_0_(*k*) and comparison sequences *y*_*i*_(*k*)  (*i* = 1,2,…, *m*; *k* = 1,2,…, *n*).

**Step 3 .**

The absolute difference sequences Δ_0*i*_(*k*) between reference sequence *y*_0_(*k*) and comparison sequences *y*_*i*_(*k*) are calculated by the formula

(2) Δ0iky0k−yik=Δi1,Δi2,…,Δin,i=1,2,…,m.

**Step 4 .**

Identify the absolute maximum Δ_max_ and minimum Δ_min_ from absolute difference sequence.

**Step 5 .**

Calculate the grey relational coefficient. The formula is

(3) $$\begin{array}{c} L_{0i}\left(\;k\;\right)=\frac{\left(\Delta_{max}+\Delta_{min}\right)}{\left(\Delta_{0i}\left(k\right)+\Delta_{max}\right)}. \end{array}$$

**Step 6 .**

Calculate correlation degree.

(4) R0ik1n∑k=1nL0ik=1nL0i1+L0i2+⋯+L0in.

### 2.2. Prediction for the Feed Grain Demand by Using Multiple Linear Regression

According to grey relational analysis, the domestic population, urbanization level, and per capita income of urban and rural residents are the main factors affecting the feed grain demand. Based on the modeling principle of multiple regression model, the linear regression model of the feed grain demand is set up, the structure form of the model [9]:

(5) $$\begin{array}{c} y_{0}=U_{0}+U_{1}x_{1}+U_{2}x_{2}+U_{3}x_{3}+\varepsilon ,\;\varepsilon \sim N\left(0,\delta^{2}\right). \end{array}$$

In the formula, *U*_1_, *U*_2_, and *U*_3_ are the undetermined parameters (regression parameters), with *ε* for unobservable random error.

### 2.3. Prediction for Main Factors That Influence the Feed Grain Demand

The ARIMA model from literature is adopted to predict the change trend of impact factors [10]. Suppose that *ω*_*t*_ is the predictive value in *t* time of various influence factors and *ω*_*t*−1_, *ω*_*t*−2_,…, *ω*_*t*−*p*_ are actual values of various impact factors in past *p* years. Setting *ω*_*t*_ = (1 − *L*)^*d*^ *y*_*t*_, among it, *y*_*t*_ is a single integer sequence with *d* order; *ω*_*t*_ is the stationary series [11]; thus the general model of the ARMA model can be expressed as

(6) ωt=φ1ωt−1+φ2ωt−2+⋯+φpωt−p+εt+θ1εt−1+⋯+θqεt−q.

In the formula, *p* and *q* are, respectively, called autoregressive order number and average order number. Suppose *L* as the lag operator; then

(7) Lωt=ωt−1,Lpωt=ωt−p.

Equation (6) can be rewritten as

(8) $$\begin{array}{c} \varphi \left(\;L\;\right)\omega_{t}=\Theta \left(\;L\;\right)\varepsilon_{t}. \end{array}$$

Among it, *φ*(*L*) = 1 − *φ*_1_ *L* − *φ*_2_ *L* ^2^ − ⋯−*φ*_*p*_ *L* ^*p*^ and Θ(*L*) = 1 + *θ*_1_ *L* + *θ*_2_ *L* ^2^ + ⋯+*θ*_*q*_ *L* ^*q*^.

ARMA(*p*, *q*) model in formula (7) can be expressed as ARIMA(*p*, *d*, *q*) after *d* order difference transformation

(9) $$\begin{array}{c} \varphi \left(\;L\;\right){\left(\;1-L\;\right)}^{d}y_{t}=\Theta \left(\;L\;\right)\varepsilon_{t}. \end{array}$$

*ε*_*t*_ is a white noise process with its mean value which is 0 and variance is *σ* ^2^ [12].

## 3. Simulation Analysis

**Figure 1:**
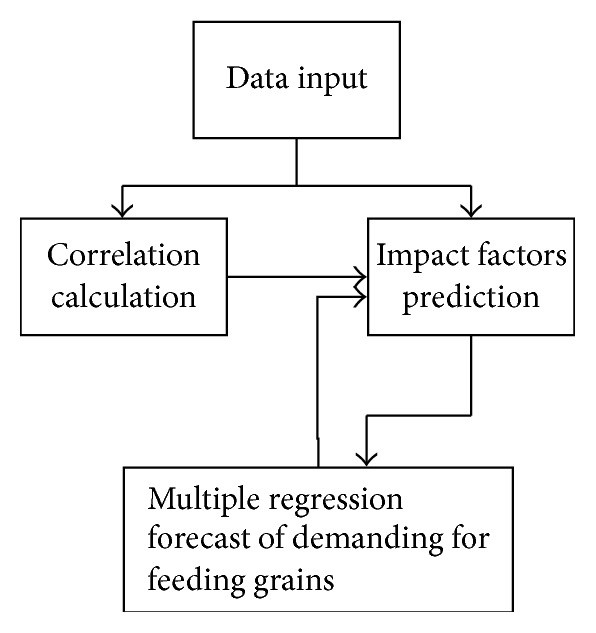
Dynamic prediction simulation process of feed grain demand.

The dynamic simulation process based on the ARIMA model and multiple regression model to predict feed grain demand is shown in Figure 1.

The dynamic prediction algorithm of feed grain demand is shown in Figure 1; define the year of 1981 as *t* = 1 and thus 2007 as *t* = 27. The feed grain demand of urban and rural population is, respectively, expressed as *y*_0_(*t*) and *y*_1_(*t*); the three factors are, respectively, defined as *x*_1_(*t*), *x*_2_(*t*), and *x*_3_(*t*). According to the simulation process shown in Figure 1, the forecast process of feed grain demand in this paper is shown in the following:

(1) When* t* = 1~27, calculate the correlation degree and relational sequence, respectively, between *y*_0_(*t*) and *y*_1_(*t*) and *x*_1_(*t*), *x*_2_(*t*), and *x*_3_(*t*).
(2) Use ARIMA model to predict *x*_1_(*t*), *x*_2_(*t*), and *x*_3_(*t*) when *t* > 27.
(3) Use multiple regression method to predict urban feed grain demand *y*_0_(*t*) (*t* = 28) and rural feed grain demand *y*_1_(*t*) (*t* = 28).
(4) Repeat (3). Urban and rural long-term prediction of feed grain demand can be completed.

### 3.1. Correlation Calculation

The data about the feed grain demand, urban and rural population, urbanization level, and urban and rural residents per capita income between 1981 and 2007 are selected from* Rural China Statistical Yearbook* [13] as the training data; meanwhile the data from 2008 to 2012 are selected as the precision test data as shown in Table 1. The feed grain demand can be got by the sum of per capita meat, egg, milk, and aquatic product consumption multiplied by the urban and rural population, respectively, and then according to the conversion ratio of feed grain to meat which is 3.7 to 1, the conversion ratio to egg which is 2.7 to 1, the conversion ratio to milk which is 0.5 to 1, and the conversion ratio to aquatic material which is 0.4 to 1 to get the final result [14, 15].

The correlation degree and relational order are obtained by using the grey correlation analysis method, while the data about the feed grain demand are calculated in Table 1 as reference sequence; at the same time urban and rural population, urbanization level, and urban and rural residents per capita income are calculated as comparative sequence. The results are shown in Table 2.

As shown in Table 2, the correlation degree and relational order of various factors which affected the urban and rural feed grain demand are not completely the same; on the basis of that, it will be able to improve the prediction accuracy by predicting towns and rural feed grain demand separately.

### 3.2. Impact Factors Prediction

ARIMA(*p*, *d*, *q*) model described in Section 2.3 is adopted to predict the three factors including urban and rural population, urbanization level, and urban and rural residents per capita income. The prediction of impact factors for urban feed grain demand in 2008 is taken as an example in this paper, and the results are shown in Table 3. The forecast data will be used to forecast feed grain demand in 2008.

### 3.3. Prediction for Feed Grain Demand by Using Multiple Regression

The multiple regression model of urban and rural demand for feed grain demands is set up, respectively, in 2008 by using EVIEWS statistical software, while three factors mentioned above are taken as independent variables and China's urban and rural residents' feed grain demand is taken as the dependent variable. The models are shown as follows:

(10) y0=−4240163+151.53x01+210419.4x02−195.0006x03,

(11) y1=−21283643+232.867x11+392070.5x12−543.328x13.

Among them, *y*_0_ and *y*_1_ represent the urban and rural feed grain demand, respectively, *x*_01_ is urban population, and *x*_11_ is rural population. *x*_02_ and *x*_12_ represent urbanization level, *x*_03_ is urban residents per capita income, and *x*_13_ is rural residents per capita income. The predicted value of three factors in 2008 was typed in (10) and (11), respectively; then the value of urban and rural feed grain demand in 2008 can be calculated; the results are 9807134 tons and 6663724.9 tons.

In the above multivariate regression model of urban and rural feed grain demand, the model prediction coefficient of different years will change dynamically as the change of correlation of feed grains and affecting factors; then it forms a dynamic forecast system.

### 3.4. Simulation Results

The value of feed grain demand in 2008–2012 can be predicted according to (10) and (11); the result is shown in Table 4. A grey forecasting model by using residual error correction on the feed grain demand in literature [16] is also given in Table 4.

From Table 4 and combined with the feed grain demand between urban and rural areas since 1981, it can be seen that the basic trend of feed grain demand overall present rises steadily [17, 18]. The feed grain demand increased by 4 times, and the average annual growth rate is 14.8% from 1981 to 2007. Analysis shows that the income level of our country residents is low, and the consumption structure is unitary, mainly grain consumption before the reform and open policy. In recent years, the demand for animal products structure is changing and it mainly displays in the increasing demand for meat, eggs, milk, and aquatic products because people's living standards have been continuously improved.

In addition, compared with the grey system model in literature [16], the joint dynamic prediction model in this paper can track the change of impact factors, so it can achieve good long-term forecasts. Meanwhile the mean relative error of proposed model is 0.46% and has higher superiority in forecasting precision compared with traditional grey forecasting model of which the mean relative error is 6.4%. It is fully illustrated that the dynamic impact factor regression analysis method used to predict the feed grain demand is feasible.

## 4. Conclusion

The dynamic influence factors in combination with multivariate regression analysis method are used in this paper to forecast the feed grain demand in China since 2008. Prediction results show that China's demand for feed grains will increase year by year in the next 10 years, and the average relative error between the actual and predicted value by using the dynamic impact factor regression model is 0.46%, superior to the traditional grey system model. At present, China's feed grain demand represents more than 30% of the total demand for grain; the proportion of which feed grain demand on total demand for grain increased year by year shows the increasing influence of feed grains on food security, so it has become a necessary work to research the feed grain demand deeply for ensuring food security.

## Figures and Tables

**Table 1:** Statistical data of various impact factors.

Year	Meat		Egg		Milk		Aquatic product		Population (ten thousand people)		Per capita income (yuan)		Urbanization level (%)
	Urban	Rural	Urban	Rural	Urban	Rural	Urban	Rural	Urban	Rural	Urban	Rural	
1981	20.5	9.4	5.2	1.3	4.1	0.7	7.3	1.3	20171	79901	500.4	223.4	14.2
1982	21	9.9	5.9	1.4	4.5	0.7	7.7	1.3	21480	80174	535.3	270.1	14.4
1983	22.5	10.8	6.9	1.6	4.6	0.8	8.1	1.6	22274	80734	564.6	309.8	14.6
1984	22.8	11.5	7.6	1.8	5.2	0.8	7.8	1.7	24017	80340	652.1	355.3	14.7
1985	24	12	8.8	2.1	6.4	0.8	7.8	1.6	25094	80757	739.1	397.6	14.8
1986	25.3	12.9	7.1	2.1	4.7	1.4	8.2	1.9	26366	81141	900.9	423.8	15
1987	25.4	12.9	6.6	2.3	5.5	1.1	7.9	2	27674	81626	1002.1	462.6	15.1
1988	23.7	12	6.9	2.3	5.1	1.1	7.1	1.9	28661	82365	1180.2	544.9	15.3
1989	23.9	12.3	7.1	2.4	4.2	1	7.6	2.1	29540	83164	1373.9	601.5	15.4
1990	25.2	12.6	7.3	2.4	4.6	1.1	7.7	2.1	30195	84138	1510.2	686.3	15.5
1991	26.6	13.5	8.3	2.7	4.7	1.3	8	2.2	31203	84620	1700.6	708.6	15.9
1992	26.5	13.3	9.5	2.9	5.5	1.5	8.2	2.3	32175	84996	2026.6	784	16.2
1993	26	13.3	8.9	2.9	5.4	0.9	8	2.8	33173	85344	2577.4	921.6	16.5
1994	24.3	12.6	9.7	3	5.3	0.7	8.5	3	34169	85681	3496.2	1221	16.8
1995	23.6	13.1	9.7	3.2	4.6	0.6	9.2	3.4	35174	85947	4283	1577.7	17.2
1996	25.8	14.8	9.6	3.4	4.8	0.8	9.25	3.7	37304	85085	4838.9	1926.1	18.4
1997	25.5	15.1	11.1	4.1	5.1	1	9.3	3.8	39449	84177	5160.3	2090.1	19.6
1998	25.5	15.5	10.2	4.1	6.2	0.9	9.84	3.7	41608	83153	5425.1	2162	20.8
1999	26.7	16.4	10.9	4.3	7.9	1	10.3	3.8	43748	82038	5854	2210.3	22
2000	25.4	18.3	11.2	4.8	9.9	1.1	11.7	3.9	45906	80837	6280	2253.4	23.2
2001	26.5	18.2	10.4	4.7	11.9	1.2	10.33	4.1	48064	79563	6859.6	2366.4	24.4
2002	32.5	18.6	10.6	4.7	15.7	1.2	13.2	4.4	50212	78241	7702.8	2475.6	25.8
2003	32.9	19.7	11.2	4.8	18.6	1.7	13.4	4.7	52376	76851	8472.2	2622.2	27.2
2004	29.3	19.2	10.4	4.6	18.8	2	12.5	4.5	54283	75705	9421.6	2936.4	28.9
2005	32.9	22.4	10.4	4.7	17.9	2.9	12.6	4.9	56212	74544	10493	3254.9	30.7
2006	32.1	22.3	10.4	5	18.3	3.1	13	5	58288	73160	11759.5	3587	32.5
2007	31.8	20.5	10.3	4.7	17.8	3.5	14.2	5.4	60633	71496	13785.8	4140.4	34.3
2008	31.2	20.2	10.7	5.4	15.2	3.4	11.9	5.2	62403	70399	15780.8	4760.6	36
2009	34.7	21.5	10.6	5.3	14.9	3.6	12.2	5.3	64512	68938	17174.7	5153.2	37.7
2010	34.7	22.2	10	5.1	14	3.6	15.2	5.2	66978	67113	19109.4	5919	38.8
2011	35.2	23.3	10.1	5.4	13.7	5.2	14.6	5.4	69079	65656	21809.8	6977.3	40.6
2012	35.7	23.5	10.5	5.9	14	5.3	15.2	5.4	71182	64222	24564.7	7916.6	42.4

Note: (1) unit: per capita consumption in kilograms; (2) the data are from *Rural China Statistical Yearbook*.

**Table 2:** The grey correlation analysis about each influencing factor in 1981–2007 of urban and rural feed grain demand.

Influencing factor	Urban		Rural	
	Correlation degree	Relational order	Correlation degree	Relational order
(Urban/rural) population	0.9370	1	0.9255	2
Urbanization level	0.9047	2	0.9641	1
(Urban/rural) per capita income	0.7236	3	0.6881	3

**Table 3:** Predicted value of various influencing factors in 2008.

Influencing factor	Model	Adjusted *R* ^2^	Predicted value in 2008
Urban population	ARIMA(3, 2, 6)	0.956	62965.45
Urbanization level	ARIMA(7, 2, 2)	0.848	36.1254
Urban residents per capita income	ARIMA(4, 2, 5)	0.876	15872.19

**Table 4:** The comparison between the actual value and predicted value of feed grain demand under different prediction models (unit: ten thousand tons).

	Year	Actual value	Predicted value	Relative error	Mean relative error
Combined dynamic forecasting model	2008	16332.1	16470.9	0.8%	0.46%
	2009	17665.2	17795.3	0.7%	
	2010	17981.0	17928.4	0.2%	
	2011	18687.2	18798.5	0.5%	
	2012	19267.6	19243.8	0.1%	

Grey forecasting model	2008	16332.1	17925.4	9.7%	6.4%
	2009	17665.2	18426.9	4.3%	
	2010	17981.0	19246.7	7.0%	
	2011	18687.2	19875.1	6.4%	
	2012	19267.6	20144.8	4.6%	
